# Compact on-Chip Temperature Sensors Based on Dielectric-Loaded Plasmonic Waveguide-Ring Resonators

**DOI:** 10.3390/s110201992

**Published:** 2011-02-07

**Authors:** Thomas B. Andersen, Zhanghua Han, Sergey I. Bozhevolnyi

**Affiliations:** Institute of Technology and Innovation, University of Southern Denmark, Niels Bohrs Alle 1, DK-5230 Odense M, Denmark; E-Mails: tbsa@iti.sdu.dk (T.B.A.); seib@iti.sdu.dk (S.I.B.)

**Keywords:** temperature sensor, dielectric-loaded surface plasmon-polariton waveguide, waveguide-ring resonator

## Abstract

The application of a waveguide-ring resonator based on dielectric-loaded surface plasmon-polariton waveguides as a temperature sensor is demonstrated in this paper and the influence of temperature change to the transmission through the waveguide-ring resonator system is comprehensively analyzed. The results show that the roundtrip phase change in the ring resonator due to the temperature change is the major reason for the transmission variation. The performance of the temperature sensor is also discussed and it is shown that for a waveguide-ring resonator with the resonator radius around 5 μm and waveguide-ring gap of 500 nm which gives a footprint around 140 μm^2^, the temperature sensitivity at the order of 10^−2^ *K* can be achieved with the input power of 100 μW within the measurement sensitivity limit of a practical optical detector.

## 1. Introduction

Temperature sensing based on optical techniques is promising and remains an area of continuing and intensive research interest around the World in recent years due to some advantages compared to other temperature measurement techniques, e.g., high sensitivity, large temperature range and the stability and immunity of optical signal to the turbulence of the environmental noises [1]. Up till now, fiber-optic temperature sensors constitute a major category of the optical temperature sensors, and they mainly employ the principles of fiber Bragg gratings [2] or surface plasmon resonance (SPR) [3]. The temperature change will introduce a noticeable shift in the central resonance wavelength, thus people can obtain the information for the temperature with high sensitivity by monitoring the resonance wavelength change. These fiber-optic temperature sensors can take the advantage of the well-developed fiber-optics technique and are very favorable for constructing remote distributed sensing networks with low propagation loss in optical fibers and wavelength division multiplexing techniques. However, all these fiber-optic temperature sensors are bulky and can hardly be used as chip scale temperature sensors. In addition, the measurement of resonance wavelength shift needs an optical spectrum analyzer, which is quite expensive and can hardly be integrated into an optical integrated circuit chip.

In this paper, we propose to realize chip scale temperature sensors based on dielectric-loaded surface plasmon-polariton waveguide-ring resonators (WRR). Surface plasmons are the surface waves due to the coupling of electromagnetic waves to the collective electron oscillations in the metals, and they propagate along the interface between the metal and the dielectrics, with the field decaying exponentially along the direction perpendicular to the interface [4], providing a new way of manipulating light at the nano scale. The light energy of surface plasmons is mainly localized at the interface between the metal and the dielectric with the major part in the dielectric, so the propagation of surface plasmons is greatly affected by the dielectric properties, which in principle implies that the use of surface plasmons in sensing is also quite promising. What’s more, the light field near the metal/dielectric is greatly enhanced and this will improve the sensing sensitivity considerably. In the literature much effort has been made to investigate the use of surface plasmons for sensing, and while some decent results have been reported [5], most of them still rely on SPRs. In the meanwhile, plasmonic waveguides have been attracting much attention in recent years owing to their ability to spatially confine light below the diffraction limit [6], thereby potentially enabling photonic device integration on a scale not accessible with conventional dielectric waveguide-based photonic integrated circuits, and this opens up a new avenue for further miniaturization of optical components. To date, various plasmonic waveguide structures have been proposed and investigated [7–9] for planar photonic integration. But the use of plasmonic waveguides in on-chip sensing has been rarely explored. Among those plasmonic waveguides proposed up till now, the dielectric-loaded surface plasmon-polariton waveguide (DLSPPW) is quite promising due to its compactness, ease of fabrication and the moderate propagation loss of surface plasmons in this waveguide. Some polymers, e.g., Poly-methylmethacrylate (PMMA), are usually employed as the dielectric material in the DLSPPW and this makes DLSPPW a good candidate for temperature sensors due to the good thermo-optic effects of the polymers.

**Figure 1.**
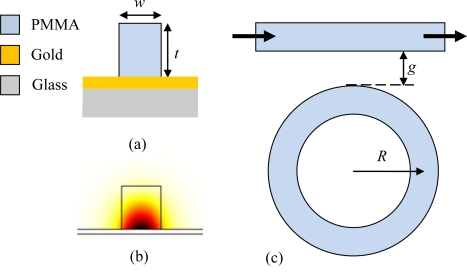
**(a)** Schematic figure for the cross section of DLSPPW; **(b)** the mode profile for the DLSPPW at the wavelength of 1.5 μm; **(c)** Schematic illustration of WRR with the dielectric-loaded surface plasmon waveguide laterally coupled to a ring resonator.

Figure 1(a) gives the schematic figure for the cross section of the DLSPPW, showing that a PMMA ridge with width 500 nm and height 550 nm is deposited onto a 60 nm thick gold film, which is supported by a thin glass substrate. In Figure 1(b) the mode profile for the 1.5 μm wavelength is also shown, from which one can see the tight lateral confinement and that the light is well confined near the bottom of the PMMA ridge in the perpendicular direction. One main advantage of the DLSPPW is that metal can be used as a negative-permittivity material to support the plasmonic mode, as well as part of the electrical circuit, so that both the optical and electrical signals can propagate through the DLSPPW at the same time. This can also be employed to heat the polymer ridges by applying some electric current through the metal strip so that the index of the polymer will be changed due to thermo-optical effect. As a consequence, the mode effective index of the DLSPPW will also change in both the real part and the imaginary part, and these two parameters will determine the power transmission through a specific waveguide. The polymer refractive index is also dependent on the environmental temperature; then one can analyze the temperature change by monitoring the power transmission. To further enhance the sensitivity of the transmission to the index change, a WRR system schematically shown in Figure 1(c) is adopted in this paper because of the high sensitivity of the transmission through the bus waveguide to the index change in the ring for some wavelengths. In the WRR system, a straight DLSPPW is laterally coupled to a ring resonator with a small gap between them and both the two components are placed on a thin gold layer. These structures can be easily fabricated with deep ultraviolet lithography or electron beam lithography techniques when the polymers can work as both the resist and the DLSPPW core material.

In the following section we will analyze in detail how the temperature change will affect the power transmission through the WRR system. Some numerical results will be given in Section 3 to show the dependence of power transmission on the temperature and the performance of this temperature sensor is also discussed. The whole paper concludes in Section 4.

## 2. Principle

It is well known that the transmission through an all-pass ring resonator schematically shown in Figure 1(c) is determined by the following Equation [10]:

(1) $$T_{r}=\frac{\alpha^{2}+\tau^{2}-2\alpha \tau \;\text{cos}(\theta )}{1+\alpha^{2}\tau^{2}-2\alpha \tau \;\text{cos}(\theta )}=\frac{\alpha^{2}+\tau^{2}-2\alpha \tau \;\text{cos}(\frac{2\pi }{\lambda }N_{\mathrm{eff}}^{\prime }\;(\lambda )2\pi R)}{1+\alpha^{2}\tau^{2}-2\alpha \tau \;\text{cos}(\frac{2\pi }{\lambda }N_{\mathrm{eff}}^{\prime }\;(\lambda )2\pi R)}$$

in which *α* = *σ* exp (−2*πRk*_0_*N″_eff_*) is the inner circulation factor describing the internal loss where σ accounts for the pure bending loss and *N″_eff_* is the imaginary part of the mode effective index *N_eff_* (*N_eff_* = *N′_eff_* + *jN″_eff_*), τ is the transmission coefficient in the bus waveguide through the waveguide-ring interaction region. θ is the round trip phase, determined by the ring radius *R* and the real part of the mode effective index *N′_eff_* (*λ*) at the free space wavelength λ. *k*_0_ = 2*π* / *λ* is the propagation constant in free space.

In Equation (1), the parameters α, τ and θ are all dependant on polymer index, so they are all functions of temperature *T* when the polymer thermo-optic effect is considered. Note that the thermal expansion is not considered here because at room temperature the linear thermal expansion coefficient of PMMA, the polymer adopted in our calculation, is about one order of magnitude smaller (3.6 × 10^−5^ /K) than the effect of temperature on refractive index (−1.05 × 10^−4^/K) [11]. As for gold, its linear thermal expansion coefficient at room temperature is even smaller (1.42 × 10^−5^/K) than that of PMMA, thus we don’t take into account in our calculations the thickness change of gold due to thermal expansion. Therefore the dimensions, including *w*, *g*, *R* and *t*, shown in Figure 1 are considered to be irrelevant to temperature *T*. Under this condition *T_r_* can be written as *T_r_* = *T_r_*(*α*(*T*), *τ*(*T*), *θ*(*T*)). Then the derivative of *T_r_* with respect to *T* is:

(2) $$\frac{{\mathrm{dT}}_{r}}{\mathrm{dT}}=\frac{{\mathrm{dT}}_{r}}{d\alpha }\frac{d\alpha }{\mathrm{dT}}+\frac{{\mathrm{dT}}_{r}}{d\tau }\frac{d\tau }{\mathrm{dT}}+\frac{{\mathrm{dT}}_{r}}{d\theta }\frac{d\theta }{\mathrm{dT}}\;□\;A+B+C$$

We will discuss each term of *A, B* and *C* separately. Using the expression for α given above one obtains:

(3) $$A=\frac{{\mathrm{dT}}_{r}}{d\alpha }\frac{d\alpha }{\mathrm{dT}}=\sigma (-2\pi {\mathrm{Rk}}_{0})e^{-2\pi {\mathrm{Rk}}_{0}N_{\mathrm{eff}}^{\prime \prime }}\;\frac{{\mathrm{dT}}_{r}}{d\alpha }\frac{{\mathrm{dN}}_{\mathrm{eff}}^{\prime \prime }}{\mathrm{dT}}=\sigma (-2\pi {\mathrm{Rk}}_{0})e^{-2\pi {\mathrm{Rk}}_{0}N_{\mathrm{eff}}^{\prime \prime }}\;\frac{{\mathrm{dT}}_{r}}{d\alpha }\frac{{\mathrm{dN}}_{\mathrm{eff}}^{\prime \prime }}{\mathrm{dn}}\frac{\mathrm{dn}}{\mathrm{dT}}$$

The last factor *dn / dT* is the thermo optical coefficient of the polymer, *dN″_eff_ / dn* describes how the imaginary part of the mode effective index changes as a function of the polymer refractive index *n* and *dT_r_* / *dα* expresses the change in transmission when the propagation loss in the ring changes.

Transmission coefficient *τ* will suffer from some change when *n* changes with temperature since the mode property will be affected and the transmission also depends on *τ*. This all together gives:

(4) $$B=\frac{{\mathrm{dT}}_{r}}{d\tau }\frac{d\tau }{\mathrm{dT}}=\frac{{\mathrm{dT}}_{r}}{d\tau }\frac{d\tau }{\mathrm{dn}}\frac{\mathrm{dn}}{\mathrm{dT}}$$

When it comes to the last term *C*, it is related to the change in the accumulated round trip phase *θ* due to variations in the real part of the mode effective index. This in turn affects the interference between the ring and the bus waveguide and consequently changes the overall transmission. We cannot measure directly how the transmission is affected by change in roundtrip phase, *i.e.*, *dT_r_* / *dθ*, but we know from our experiments how transmission changes with wavelength *dT_r_* / *dλ*. Note that in this paper, we are investigating changes in transmission *versus* temperature at a certain wavelength *λ*_0_. So we can get:

(5) $$C=\frac{{\mathrm{dT}}_{r}}{d\theta }\frac{d\theta }{dT}=(\frac{{\mathrm{dT}}_{r}}{d\lambda }\frac{1}{\frac{d\theta }{d\lambda }}){|}_{\begin{array}{l} d\theta \to 0\\ \lambda =\lambda_{0} \end{array}}\left(\frac{d\theta }{{\mathrm{dN}}_{\mathrm{eff}}^{'}}\frac{{\mathrm{dN}}_{\mathrm{eff}}^{'}}{\mathrm{dT}}\right){|}_{\begin{array}{l} dT\to 0\\ \lambda =\lambda_{0} \end{array}}$$

Since *θ* is a function of *N′_eff_* at *λ*_0_, the connection between *dθ* and *dN′_eff_* can be derived mathematically. With *θ* = 4*π*^2^ *RN′_eff_* / *λ*, we obtain that *dθ* = 4*π*^2^ *R*(*λdN′_eff_* – *N′_eff_ dλ*) / *λ*^2^. Because *dθ* → 0 at *λ*_0_, it can be concluded from *θ* = 4*π*^2^ *RN′_eff_* / *λ* that *dN′_eff_* → 0, then we have *dθ* / *dλ* = −4*π*^2^ *RN′_eff_* / *λ*^2^. With the expression for *θ*, we also have *dθ* / *dN′_eff_* |_*λ*=*λ*_0__ = 4*π*^2^ *R / λ*. As for the term *dN′_eff_ / dT* describing mode effective index change with temperature, we realize it’s not possible to calculate it directly, but we can determine how the mode effective index changes with polymer change index of refraction *n* and it’s known how *n* changes with temperature: *dN′_eff_ / dT* = (*dN′_eff_ / dn*)(*dn / dT*). This together gives the result:

(6) $$C=-\frac{{\mathrm{dT}}_{r}}{d\lambda }\frac{\lambda }{N_{\mathrm{eff}}^{\prime }}\frac{{\mathrm{dN}}_{\mathrm{eff}}^{\prime }}{\mathrm{dn}}\frac{\mathrm{dn}}{\mathrm{dT}}$$

So in principle the polymer index change due to the thermo-optic effect will cause some changes in α, τ and θ [or *N′_eff_* (*λ*)], which further lead to the variation of power transmission *T_r_* through the WRR. Thus the temperature change can be calculated via the *T_r_* variation. This is the basic idea of the temperature sensor based on dielectric-loaded plasmonic waveguide-ring resonators.

## 3. Results and Discussion

In the previous section, we have shown analytically the temperature dependence of the transmission output through a WRR system. In this section, we will discuss quantitatively the three terms of *A*, *B* and *C* for a specific WRR system. That is, how the changes of α, τ and θ due to the change of polymer index as a function of temperature will affect the transmission through the WRR.

**Figure 2.**
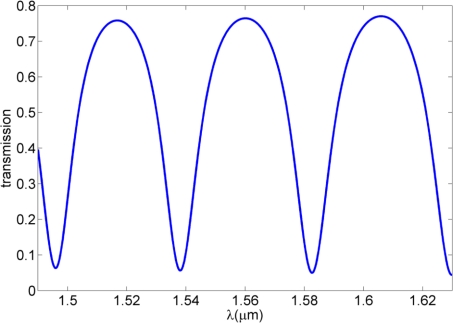
Transmission spectrum for an all-pass ring resonator based on DLSPPW.

We start with a specific example of a WRR with the ring radius *R* equaling to 5.39 μm and waveguide-ring gap *g* being 0.5 μm, whose transmission spectrum is shown in Figure 2. Here it is assumed that the pure bending loss σ is 0.71 and the transmission coefficient τ is 0.66. In the calculations of the mode effective index, *i.e.*, *N′_eff_* and *N″_eff_*, effective index method [12] is adopted to have a fast and simple calculation over this wavelength range while retaining an acceptable accuracy compared to the pure numerical method, e.g., the finite-element method (FEM) based mode solvers. We used the *N′_eff_* to be −2.52 × 10^5^m^−1^λ + 1.6138 and the power propagation length *L_sp_*(*λ*), which is defined as *L_sp_*(*λ*) = *λ* / (4*π N″_eff_*), to be 71.2λ − 60.88 μm. Note that the equations for *N′_eff_* and *L_sp_*(*λ*) will be dependent on the geometry of the DLSPPW show in Figure 1(a). All the assumptions are proven to be valid because they give a good agreement between the experimental transmission result and the analytical result from Equation (1) [13]. From Figure 2 one can see that the transmission exhibits period dips with extinction ratios above 10 dB and the free spectral range around 45 nm.

With this ring resonator as an example, the specific values of *A*, *B* and *C* in Equation (2) will be approximately estimated for some wavelengths. We will start with *C* first, because it is strongly affected by the working wavelength due to the term of *dT_r_/dλ*. Taking the derivative of *T_r_* with respect to λ, a maximum of *dT_r_/dλ* with the value 7.394 × 10^7^ is found at the wavelength around 1.5 μm. Assuming the complex refractive index of gold to be the experimental value of 0.53 + 9.51j [14] and the PMMA index to be 1.493, using a commercial FEM mode solver of Comsol Multiphysics, the mode effective index for the DLSPPW schematically shown in Figure 1(a) is found to be 1.224 + 0.00384j. For the calculation of *dN′_eff_* / *dn*, the index of PMMA is changed by a small amount Δ*n* and FEM mode solver is used again to find the new value of *N′_eff_*. The PMMA index change with respect to temperature *dn/dT* is −1.05 × 10^−4^ /*K* [11]. Then the specific value of *C* at around 1.5 μm is estimated to be *C* = 8.4 × 10^−3^ /*K*.

For the calculation of *A* at 1.5μm, first *α* is found at this wavelength, with which *T_r_* is further calculated according to Equation (1). Using a similar approach to that described above, *α* is changed by a small amount Δ*α* and *T_r_* is calculated again, then *dT_r_* / *dα* is obtained. With respect to *dN″_eff_* / *dn* calculation, FEM mode solver is also used twice with a small change of the PMMA index, then the value of *A* is estimated as *A* = 9.3 × 10^−6^ /*K*. The calculation of *B* is not so straightforward because the determination of transmission coefficient τ usually needs a full wave simulation. To have a rough estimation of it, we switch to a simpler method. As is known, the coupling of optical power from one straight waveguide to the other straight waveguide is determined by their interaction length *l* and the coupling length *L_c_* between the two waveguides, while the latter is the length over which the power can be completely transferred from one waveguide to the other and *L_c_* can be calculated using *L_c_* = *λ* / (2|*n_e_* – *n_o_*|) where *n_e_* and *n_o_* are the mode effective index for the even mode and the odd mode respectively for the two-waveguide system, so the transmission coefficient τ can be roughly obtained by *τ* = cos (*π* / 2×*l* / *L_c_*) [15]. With the FEM mode solver and having two DLSPPW with a gap *g* of 500 nm in the structure, both *n_e_* and *n_o_* can be obtained with which *L_c_* can be calculated at 1.5 μm. Assuming the interaction length between the straight waveguide and the ring resonator to be *l* = 7.15 μm, we can get the transmission coefficient τ to be about 0.66, which agrees quite well with the value of τ used to generate Figure 2. By changing the PMMA index with a small amount and repeating the above procedures, the dependence of τ on PMMA index *dτ* / *dn* can be calculated. Using this method, we can roughly estimate *B* at the wavelength of 1.5 μm to be *B* = −2.12×10^−4^ / *K*.

Having a comparison of *A*, *B* and *C*, one can see that *A* and *B* are 2 orders and 1 order of magnitude smaller than *C*, respectively, which implies that we can ignore *A* and *B* just to simplify the analysis. These values are obtained at a wavelength when *dT_r_* / *dλ* is large, however. Note that *A* and *B* are not as sensitive to the wavelength as *C* is, so we can conclude that *dT_r_* / *dT* has its maximum when *dT_r_* / *dλ* is at its maximum.

*C* demonstrates the dependence of transmission on the roundtrip phase of the resonator waveguide, which is actually the dependence of transmission on the mode effective index. One can easily understand why *C* is much larger than *A* and *B* because the transmission through an all-pass resonator is actually due to the interference of light through the straight waveguide directly and the light coupled to the straight waveguide from the resonator after one roundtrip of propagation. The interference is very sensitive to the phase change inside the ring resonator.

So in principle the temperature sensor is mainly based on the temperature dependence of PMMA index, which further determines the mode effective index of the ring waveguide and the roundtrip phase of the ring. One may also raise the question about the influence of thermo-optic effect in the gold layer to the transmission. This can be estimated using Equations (2 ∼ 4) and (6), with *dN′_eff_* / *dn*, *dN″_eff_* / *dn*, *dτ* / *dn*, and *dn* / *dT* changed to be *dN′_eff_* / *dn_Au_*, *dN″_eff_* / *dn_Au_*, *dτ* / *dn_Au_*, and *dn_Au_* / *dT*, where *n_Au_* is the complex refractive index of gold. Our numerical calculation results show that the influence to the transmission through the WRR system due to the thermo-optic effect of gold is roughly two orders of magnitude smaller than that due to the thermo-optic effect of PMMA. Here the dependence of both of the real and imaginary parts of the complex refractive index of gold is evaluated with the temperature dependent Drude model [16]. Then for the sake of simplicity, we can only concentrate on the thermo-optic effect of PMMA in this paper.

From Equation (6), one can see that *dT_r_* / *dλ* plays a very important part in the determination of *C*. In order to have an optimum performance for the temperature sensor, one needs to have to have *dT_r_* / *dλ* as large as possible. *dT_r_* / *dλ* can be roughly estimated as 0.5(*T*_*r*max_ – *T*_*r*min_) / *λ_FWHM_* where *T*_*r*max_ and *T*_*r*min_ are the maximum and minimum transmissions respectively, and *λ_FWHM_* is the bandwidth at resonance wavelength *λ_m_*. Since *λ_FWHM_* = *λ_m_* / *Q* and the quality factor Q is limited by the low intrinsic quality factor of the plasmonic ring resonator, we need to have a large difference between *T*_*r*max_ and *T*_*r*min_ or in other words a large extinction ratio in order to increase *dT_r_* / *dλ*. As discussed in [17], for plasmonic resonators, the loss in the resonators is relatively large, *i.e.*, α is quite small. In order to have a coupling close to the critical coupling, the gap between the bus waveguide and the ring resonator should be small enough. As for the ring radius, since it determines both the roundtrip propagation loss inside the ring and the bending loss and there should be a compromise between the two, one needs to design the ring resonators carefully under specific conditions.

According to the discussions shown above, the sensitivity of transmission to the temperature is on the order of 10^−3^ / *K*. In practice, the minimal detectable temperature change is affected by the optical detector sensitivity, and can be described as:

(6) $${\mathrm{dT}}_{\text{min}}=\mathrm{NEP}\times \sqrt{B}/(P_{\mathrm{in}}k_{\mathrm{loss}}\;\frac{{\mathrm{dT}}_{r}}{\mathrm{dt}})$$

where *NEP* is the Noise-equivalent power of the optical detector, *B* is the bandwidth of the detector, *P_in_* is the input power from the light source and *k_loss_* characterizes the power loss from the light source to the detector, here considered to be fiber-coupled. If we use the *NEP* to be 1.4×10^−12^ $W\sqrt{\mathrm{Hz}}$ and *B* to be 320 kHz, which are the characteristics of a commercially available optical detector from Thorlabs (PDA10CS-EC), and assume *k_loss_* to be 3.8 × 10^−2^ [18] and the input power to be 100 μW, we can find that the minimal detectable temperature change can be as low as 2.5 × 10^−2^ K with the value of *dT_r_* / *dT* equaling to 8.4×10^−3^ / *K*. The sensitivity can be further increased if a lock-in-amplifier is used. In this case B can be replaced with 1/(2τ), where τ is the integration time. For τ equaling to 60 s, the sensitivity becomes as low as 4.0 × 10^−6^ K. Note the optical detector for the DLSPPW can also be replaced with a power monitor that was proposed for the long range surface plasmon polariton waveguides [19], then both the temperature sensor and the power monitor can be integrated on a single chip.

Although the change in power transmission is monitored in this temperature sensor, we can also compare the refractive index sensitivity with other temperature sensors measuring the resonance wavelength shift. Using the data in Section 3, we can find the refractive index sensitivity to be *dλ / dn* = *dλ / dN′_eff_* × *dN′_eff_ / dn* = *λ* / *N′_eff_* × *dN′_eff_* / *dn* =1090*nm* / *RIU*. And note that the sensing part alone only has a footprint of around 140 μm^2^ and is quite compact.

## 4. Conclusions

We have demonstrated in this paper the application of a WRR based on DLSPPW as a temperature sensor. The temperature dependence of transmission coefficient in the bus waveguide through the waveguide-ring interaction region, the intrinsic loss and the roundtrip phase inside the ring, as well as the influence of these parameters to the transmission through the WRR are analyzed. Theoretical calculations also show that for a WRR with the resonator radius around 5 μm and waveguide-ring gap of 500 nm, the temperature sensitivity at the order of 10^−2^ *K* can be achieved with the input power of 100 μW within the sensitivity limit of a practical photodetector. This temperature sensor is very promising as an on-chip sensor of temperature due to the compact size and high sensitivity. We believe that it will find broad applications in many areas.
